# Impact of Alkyl Polyglucosides Surfactant Lutensol GD 70 on Modification of Bacterial Cell Surface Properties

**DOI:** 10.1007/s11270-015-2327-4

**Published:** 2015-02-24

**Authors:** Wojciech Smułek, Ewa Kaczorek, Agnieszka Zgoła-Grzeskowiak, Zefiryn Cybulski

**Affiliations:** 1 Institute of Chemical Technology and Engineering, Poznan University of Technology, Berdychowo 4, 60-965 Poznan, Poland; 2 Institute of Chemistry and Technical Electrochemistry, Poznan University of Technology, Berdychowo 4, 60-965 Poznan, Poland; 3 Department of Microbiology, Greater Poland Cancer Centre, Garbary 15, 61-866 Poznan, Poland

**Keywords:** Alkyl polyglucosides, Bioavailability, Hydrophobicity, Lutensol GD 70, Surfactant biodegradation, Zeta potential

## Abstract

Alkyl polyglucosides, due to their low toxicity and environmental compatibility, could be used in biodegradation of hydrophobic compounds. In this study, the influence of Lutensol GD 70 on the cell hydrophobicity and zeta potential was measured. The particle size distribution and surfactant biodegradation were also investigated. *Microbacterium* sp. strain E19, *Pseudomonas stutzeri* strain 9, and the same strain cultivated in stress conditions were used in studies. Adding surfactant to the diesel oil system resulted in an increase of the cell surface hydrophobicity and the formation of cell aggregates (a high polydispersity index). The correlation between cell hydrophobicity and zeta potential in examined samples was not found. The results showed a significant influence of Lutensol GD 70 on the changes in cell surface properties. Moreover, a high biodegradation of a surfactant (over 50 %) by tested strains was observed. The biodegradation of Lutensol GD 70 depends on the length of both polar and nonpolar chains. A long-term contact with diesel oil of stressed strain modifies not only cell surface properties but also its ability to a surfactant biodegradation.

## Introduction

Alkyl polyglucosides, such as Lutensol GD 70, are nonionic surfactants widely used in industry to form microemulsions, such as all-purposes detergents, cleaners, and personal care products. They are made of natural occurring and renewable materials: carbohydrates and fatty alcohols (von Rybinski and Hill 1998; El-Sukkary et al. 2008). Due to their low toxicity and high biodegradability, they are an environmentally friendly alternative to the conventional surfactants (Bastl-Borrmann and Kroh 2001). Moreover, the addition of these surfactants is one of the possible methods of stimulation of hydrocarbons biodegradation (Edwards et al. 2003).

A lot of hazardous and persistent organic pollutants are hydrophobic and have low solubility in water. Surfactants can increase their solubility and improve a mass transfer between solid and aqueous phases (Zhou and Zhu 2007). On the other hand, surfactants can change the surface properties of bacterial cells, which improves their adhesion to hydrophobic surfaces (Abbasnezhad et al. 2011). Hence, hydrophobic pollutants’ degradation ability of the bacteria, may be linked to the cell surface properties (Kumari et al. 2012), but not necessarily (Owsianiak et al. 2009). Thus, a surfactant-enhanced remediation (SER) can be a promising method for the remediation of contaminated soils, groundwater, and industrial waste waters (Zhou and Zhu 2007). The determination of cell surface properties, cell adhesion, and cell barrier permeability are also important in another research areas, e.g., in the processes of metals and concrete corrosion, in food industry and in medicine (including cancer diseases diagnosis and treatment) (Harimawan et al. 2013; Hwang et al. 2012; Bañobre-López et al. 2013; Borghi et al. 2011; Hojan and Milecki 2013). In the studies on bacterial adhesion, the hydrophobicity of cell surface is a very often studied property. In order to determine it, several techniques are used, e.g., hydrophobic interaction chromatography, measurement contact angle (van Loosdrecht et al. 1987; Zita and Hermansson 1997), and the method is of microbial adhesion to hydrocarbons—MATH (Rosenberg et al. 1980), which is based on the distribution of cell population between organic and aqueous phases. The more cell is adsorbed in organic phase, the greater hydrophobicity is.

Another property depending on the cell surface properties is zeta potential, usually used in reference to colloids, and it is also an important factor in microbial adhesion. It is possible that the charge neutralization is connected to the enhanced adhesion to the oil interface (Abbasnezhad et al. 2011; Hermansson 1999). The influence of synthetic and natural surfactants on cell surface properties, during biodegradation of hydrophobic pollutants, were widely described (Das and Mukherjee 2007; Feng et al. 2013; Singh and Cameotra 2014; Wang et al. 2013; Li and Zhu 2012). But only a few publications about application of alkyl polyglucosides in bioremediation have been issued (Sałek et al. 2013).

The aim of this study was to determine the changes of cell surfaces properties in the presence of Lutensol GD 70. The differences in hydrophobicity and zeta potential between culture with and without diesel oil were examined. The correlation between these two parameters was also analyzed. Moreover, the subject of the studies was also the biodegradation of Lutensol GD 70 (Table 1), because, for the purpose of bioremediation, the surfactants used should be also biodegradable.

**Table 1:** Parameters of mass spectrometric detection characteristic to particular analytes

Glucoside	Precursor ion [M + NH_4_]^+^ (*m*/*z*)	MRM transitions (precursor ion *m*/*z* → product ion *m*/*z*)			
		MRM 1	Collision energy (V)	MRM 2	Collision energy (V)
Lutensol GD 70					
C_4_Glu	254.5	254.5 → 163.4	13	254.5 → 145.4	17
C_4_Glu_2_	416.5	416.5 → 163.4	21	416.5 → 145.4	28
C_4_Glu_3_	578.5	578.5 → 163.4	30	578.5 → 145.4	37
C_4_Glu_4_	740.5	740.5 → 163.4	41	740.5 → 145.4	51
C_10_Glu	338.5	338.5 → 163.4	15	338.5 → 145.4	19
C_10_Glu_2_	500.5	500.5 → 163.4	23	366.5 → 145.4	29
C_10_Glu_3_	662.5	662.5 → 163.4	30	662.5 → 145.4	38
C_10_Glu_4_	824.6	824.6 → 163.4	41	824.6 → 145.4	48

*MRM 1* analytical multiple reaction monitoring transition, *MRM 2* confirmatory multiple reaction monitoring transition

## Materials and Methods

### Microorganisms

The bacterial strains *Pseudomonas stutzeri* strain 9 (JN006140.1) and *Microbacterium sp.* strain E19 (JQ268557) used in the experiments were isolated from soil samples. They were denoted as Ps9 and E19. The strains were kept on plates with Mueller–Hinton agar. In order to determine the influence of diesel oil, the *P. stutzeri* strain 9 was kept on agar plates with 50 μL of diesel oil, as the only carbon and energy source, for 24 months (subcultured every month to agar plates with mineral medium and diesel oil), and was denoted as Ps9s.

### Growing Conditions

The culture mineral salts medium, used throughout these studies, was as described previously (Kaczorek et al. 2010). A liquid culture was started by adding a loopful of cells from an agar plate into a 250 mL Schott Duran® laboratory glass bottles containing 50 mL of medium. After approximately 24 h, 3–5 mL of this liquid culture was used for the inoculation of the final culture to reach an optical density (measured at 600 nm) ca. 0.1.

### Particle Size Distribution

The particle size distribution of the examined cultures was determined by applying the noninvasive back light scattering method. The Zetasizer Nano ZS apparatus (Malvern Instruments Ltd., UK) was used for this purpose. Based on the obtained results, a polydispersity index (PdI) was automatically calculated.

### Cell Surface Hydrophobicity

The modified method of microbial adhesion of hydrocarbon (Górna et al. 2011) was used to determine the cell surface hydrophobicity. In bacterial cultures, as the carbon and energy sources were used: fructose, diesel oil, heptane, toluene, Lutensol GD 70 (in different concentrations 6, 60, 120, 240, and 360 mg L^−1^). Moreover, the strains grown on diesel oil and Lutensol GD 70 were also investigated. The bacterial cells from the 7-day cultures were centrifuged (8000 rpm, 5 min, 10 °C) and washed twice with PUM buffer (19.7 g L^−1^ K_2_HPO_4_, 7.26 g L^−1^ KH_2_PO_4_, 1.8 g L^−1^ H_2_NCONH_2_, 0.2 g L^−1^ MgSO_4_ · 7H_2_O) in order to remove residual surfactant and carbon sources. Then, the cells were resuspended in the PUM buffer, an optical density was fitted to ca. 1.0. The optical density was measured at 600 nm (OD_600_) on a Shimadzu UV–Visible Spectrophotometer. Afterwards, 0.5 mL of heptane was added to 5 mL of cell suspension and vortexed for 2 min. After 60 min, the OD_600_ of the aqueous phase was measured. The microbial adhesion to hydrocarbons was calculated as follows:

$$ H = \left(1-\left({A}_1/{A}_0\right)\right) \times 100\ \% $$

*H*—hydrophobicity [%]; *A*_0_—OD_600_ of initial aqueous phase [−]; *A*_1_—OD_600_ of aqueous phase after mixing with hexadecane [−].

### Zeta Potential

The zeta potential was calculated by using the Zetasizer Nano ZS apparatus (Malvern Instruments Ltd., UK) from the Smoluchowski equation (Miyake et al. 1990), following measurements of electrophoresis mobility. The bacterial strains were grown on different carbon sources: fructose, diesel oil, heptane, toluene, Lutensol GD 70 (in different concentrations 6, 60, 120, 240, and 360 mg L^−1^) and in systems with diesel oil and Lutensol GD 70. The bacterial cells from the 7-day cultures were centrifuged (8000 rpm, 5 min, 10 °C) and washed twice with PUM buffer (19.7 g L^−1^ K_2_HPO_4_, 7.26 g L^-1^ KH_2_PO_4_, 1.8 g L^−1^ H_2_NCONH_2_, 0.2 g L^−1^ MgSO_4_ · 7H_2_O) in order to remove residual surfactant and carbon sources. Then, the cells were resuspended in the PUM buffer, and OD_600_ were fitted to ca. 1.0 (10^8^ cfu mL^−1^).

## Results and Discussion

### Particle Size Distribution

**Fig. 1:**
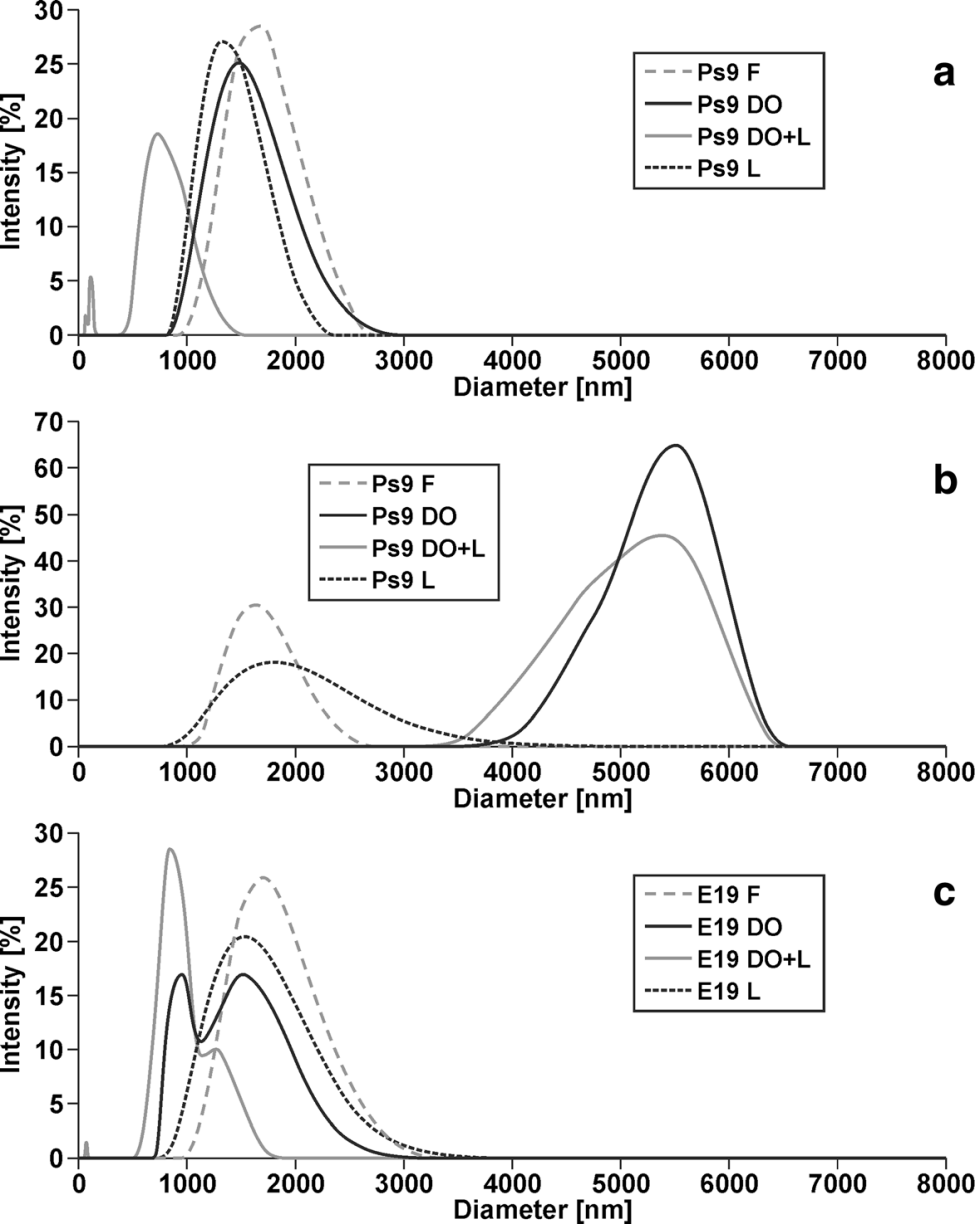
Particle size distribution during: **a** *Pseudomonas stutzeri* strain 9 (Ps9), **b** stressed *Pseudomonas stutzeri* strain 9 (Ps9s), **c** *Microbacterium* sp. strain E19 (E19) growth on a different carbon sources: *F*—with fructose, *DO*—with diesel oil, *DO + L*—with diesel oil and Lutensol GD 70 (120 mg L^−1^), *L*—with Lutensol GD 70 (120 mg L^−1^). For each sample, three measurements were made, the accuracies of the measurements being ±0.01 mV

The particle size distribution for all tested strains is shown in Fig. 1a–c. In the case of the nonstressed strains (Ps9 and E19), there was no significant difference in distribution spectra between cultures with fructose, diesel oil, and the surfactant only. In the presence of diesel oil and Lutensol GD 70, the particles are minor and two groups can be distinguished—about 100 and 700 nm. For the stressed strain (Ps9s), the particle size distributions are similar in cultures with fructose and Lutensol GD 70, and other with diesel oil. The addition of the surfactant had no influence. According to polydispersity index (PdI), the most homogeneous samples are the ones with Lutensol GD 70 only (for Ps9—0.031, for Ps9s—0.218, for E19—0.201). For each strain, the samples with Lutensol GD 70 and diesel oil as well (for Ps9—0.728, for Ps9s—1.000, for E19—0.427) are the less homogeneous.

### Changes in Cell Surface Hydrophobicity

**Fig. 2:**
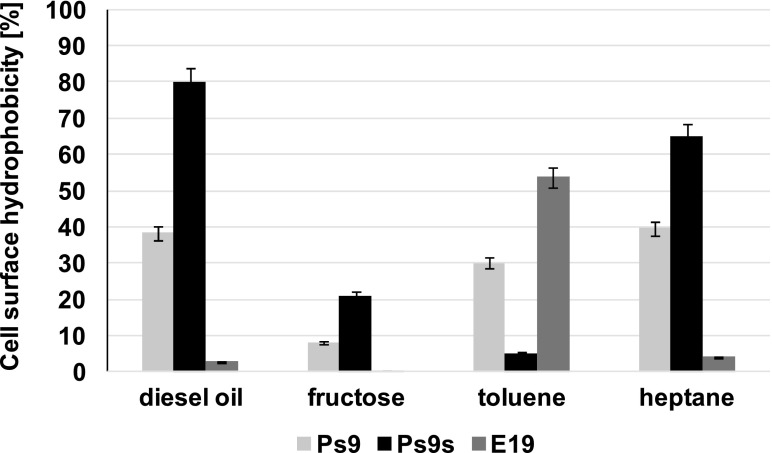
Bacterial adhesion to hydrocarbon for *Pseudomonas stutzeri* strain 9 (PS9), the same strain grown in stress conditions (PS9s) and *Microbacterium* sp. strain E19 (E19) after growth on different carbon sources: diesel oil, fructose, toluene, and heptane. Process was carried out at 25 °C for 7 days. The results have an absolute (100 %) quantitative value. Each value is the average of triplicate determinations with the standard deviation in the range of ±1.8 %

The cell surface hydrophobicity of examined strains strongly depends on the carbon source used (Fig. 2). In the presence of easily acceptable compounds, such as fructose, hydrophobicity of all strains did not exceed 20 %. In the samples from cultures with hydrocarbons, the hydrophobicity increased but it depended on the tested strain. Hydrophobicity of *P. stutzeri* (Ps9) increased in the presence of diesel oil, toluene, and heptane, and reached values between 30 and 40 %. The hydrophobicity of the same strain grown in stress conditions (Ps9s) reached 80 % in the presence of diesel oil and 65 % in heptane, but it was very low in the presence of toluene. It can suggest that in stress conditions, it has adapted more to aliphatic hydrocarbons, than to aromatic compounds like toluene. *Microbacterium* sp. strain E19 had very low hydrophobicity (less than 10 %) in all samples except for the one with toluene.

**Fig. 3:**
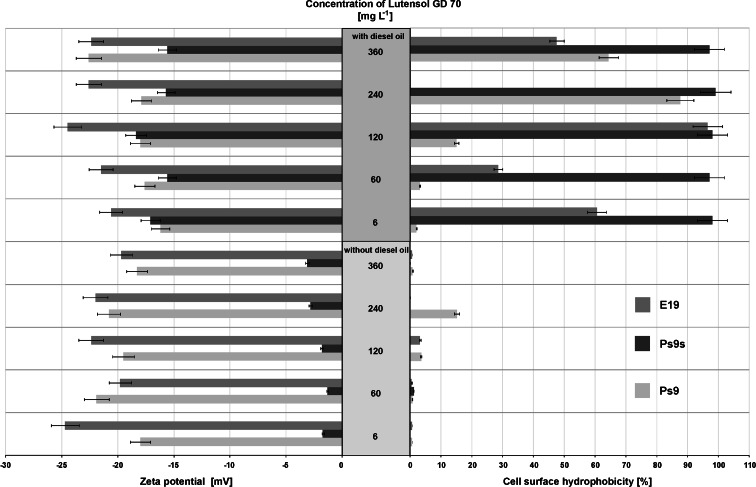
Bacterial adhesion to hydrocarbon and zeta potential for *Pseudomonas stutzeri* strain 9 (Ps9), the same strain grown in stress conditions (Ps9s) and *Microbacterium* sp*.* strain E19 (E19) after growth on Lutensol GD 70 and diesel oil (DO) with Lutensol GD 70. The concentration of diesel oil was 2 % (*w*/*v*) and surfactant 6, 30, 60, 120, 240, and 360 [mg L^−1^]. The results have an absolute (100 %) quantitative value. Process was carried out at 25 °C for 7 days. Each value is the average of triplicate determinations with the standard deviation in the range of ±1.8 % (for bacterial adhesion) and ±0.01 mV (for zeta potential)

The influence of Lutensol GD 70 on the cell surface hydrophobicity is very significant (Fig. 3). For all strains in cultures with a surfactant, only the strains with hydrophilic properties (less than 20 %) were dominating, even compared to cultures with fructose. In the presence of diesel oil and a surfactant, the hydrophobicity of all strains grew up and for Ps9s were nearly 100 %. However, the correlation between the surfactant concentration and the value of hydrophobicity was not observed.

The results observed differ from those obtained by other researchers. The addition of synthetic surfactants, like Triton X-100 and Tween 80, causes an increase of the cell surface hydrophobicity for *Pseudomonas aeruginosa* (Yuan et al. 2007). A similar effect was observed for Tween 80 or sodium dodecyl benzene sulfonate and *Citobacter* sp. SA01 (Li and Zhu 2012). On the other hand, Feng et al. (2013) found that rhamnolipid and tergitol increased *Pseudomonas putida* 852 hydrophobicity, but decreased the hydrophobicity of *Rhodococcus erythropolis* 3586. The first strain in culture without surfactants was hydrophilic, the second—hydrophobic. The differences can be caused by different surfactant adsorption mechanisms.

### Changes in Zeta Potential

**Fig. 4:**
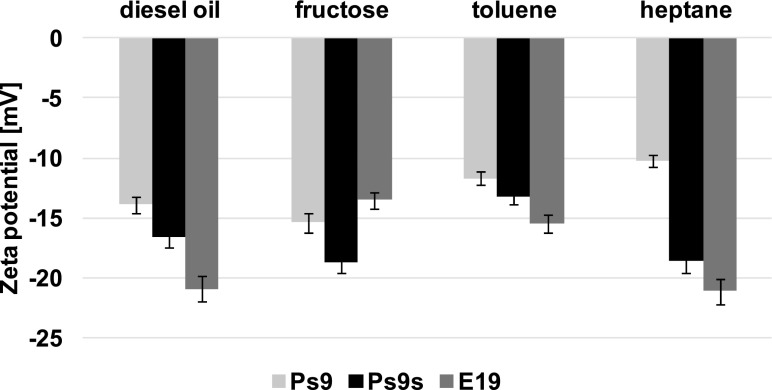
Zeta potential for *Pseudomonas stutzeri* strain 9 (Ps9), the same strain grown in stress conditions (Ps9s) and *Microbacterium* sp. strain E19 (E19) after 7 days of experiment carried out with different carbon sources: diesel oil, fructose, toluene, and heptane. For each sample, three measurements were made, the accuracies of the measurements being ±0.01 mV

The zeta potential in samples with different carbon sources for all strains fluctuated between −10 and -22 mV (Fig. 4). For *Microbacterium* sp. strain E19, the value of zeta potential was lower in cultures with hydrocarbon than in cultures with fructose, but there is no similar correlation for another strains.

The addition of a surfactant emphatically changed the zeta potential (Fig. 3), especially for Ps9s strain in cultures with surfactant only. In these samples, the zeta potential increased to more than −5 mV. It is possible that it was caused by the changes in cell well structure in stress conditions. On the other hand, a decrease of this parameter was observed for Ps9 strain. For all strains in systems with diesel oil and a surfactant, the values fluctuated between −15 and −25 mV.

Comparing with the results for hydrocarbon degrading strains *Burkholderia cepacia* and *Burkholderia multivorans* grown with Triton X-100, Igepal CA 630, and Tween 80 (Mohanty and Mukherji 2012), it can be also noticed that the addition of a surfactant changes the zeta potential of the cell surface but they depend on tested strain and surfactant as well. The influence of biosurfactants like rhamnolipids on zeta potential during diesel oil biodegradation was also observed (Zhong et al. 2007). The similar effect of reducing zeta potential by Lutensol GD 70 for strain Ps9s was observed in system with *Pseudomonas fluorescens* strain LP6a and some cationic surfactants (Hermansson 1999).

**Fig. 5:**
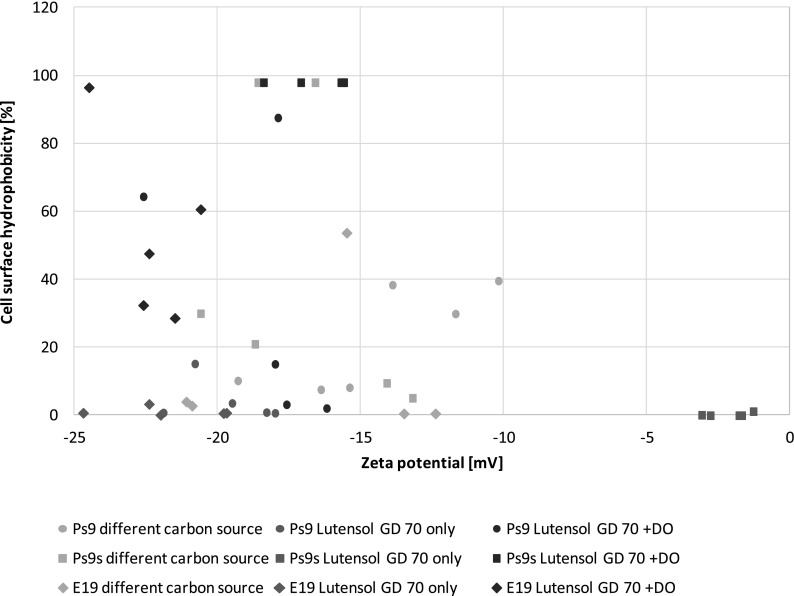
Correlation between cell surface hydrophobicity and zeta potential for *Pseudomonas stutzeri* strain 9 (Ps9), the same strain grown in stress conditions (Ps9s) and *Microbacterium* sp. strain E19 (E19)

Moreover, in Fig. 5, a relationship between two measured cell surface properties—hydrophobicity and zeta potential, is shown. In general and in any specified set of data, no correlation has been observed.

### Surfactant Biodegradation

**Fig. 6:**
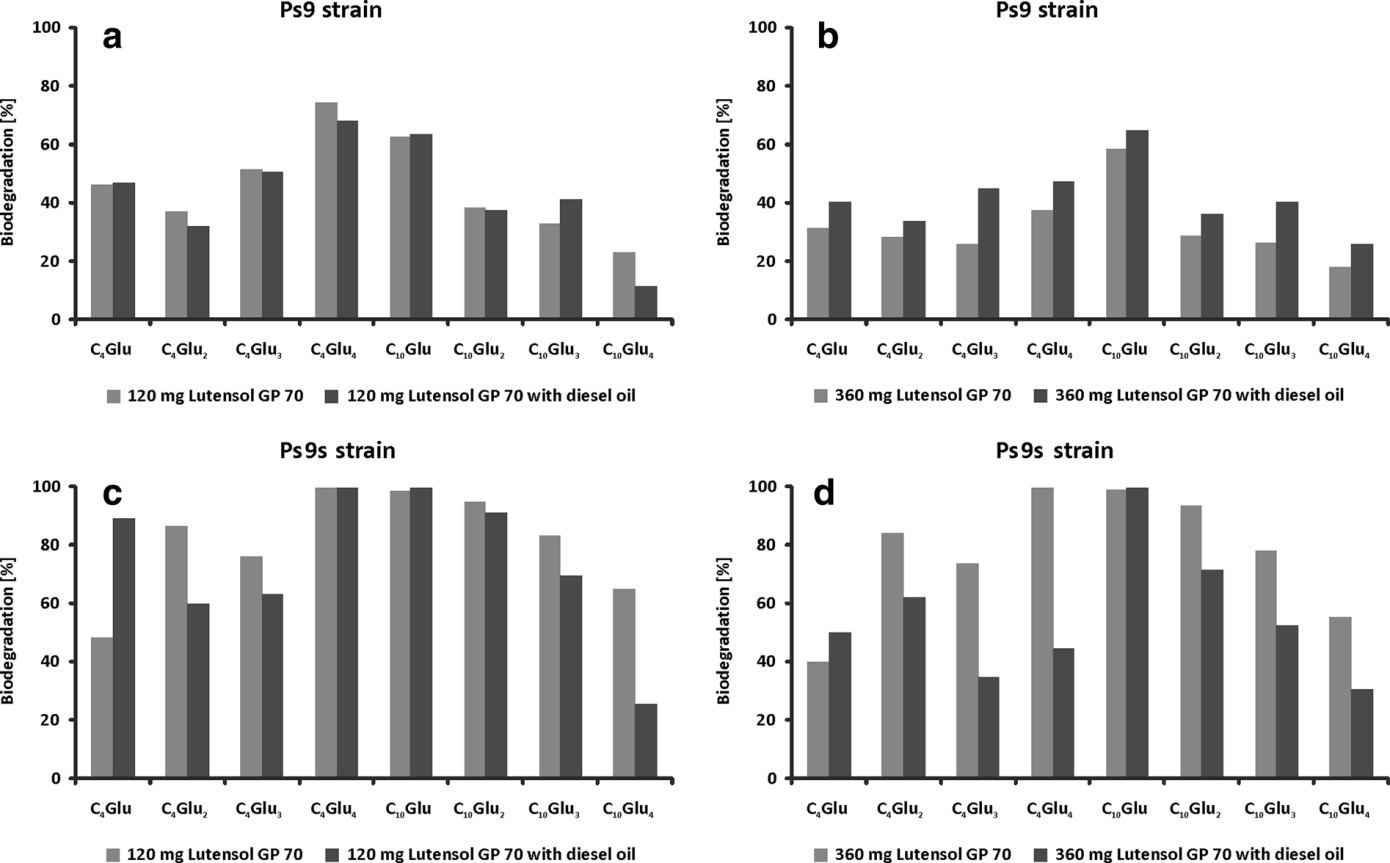
Biodegradation of Lutensol GP 70 with and without diesel oil using: **a**, **b** *Pseudomonas stutzeri* strain 9 (Ps9) and **c**, **d** the same strain grown in stress conditions (PS9s). The alkyl polyglucosides concentration was 120 and 360 mg L^−1^ and diesel oil 2 % (*w*/*v*). Process was carried out at 25 °C for 7 days

The biodegradation of Lutensol GD 70 by tested bacterial strains depends on the length of both polar and nonpolar chains. However, in most tests, no considerable dependence between the biodegradation and the length of alkyl or glucose chain can be found. The biodegradation of Lutensol GD 70 by the Ps9 strain is not high at both concentrations of this surfactant, i.e., 120 and 360 mg L^−1^ (Fig. 6a, b). Moreover, the increased amount of Lutensol GD 70 led to its lower biodegradation. On the other hand, the addition of diesel oil to the samples had no influence on the sample containing 120 mg L^−1^ of Lutensol GD 70, and only a small increase of biodegradation was observed for the sample containing 360 mg L^−1^ of Lutensol GD 70.

The biodegradation of Lutensol GD 70 by the stressed Ps9 strain was relatively high at both concentrations of this surfactant (Fig. 6c, d). There was no difference between samples containing 120 and 360 mg L^−1^ of Lutensol GD 70. The addition of diesel oil had no influence on the biodegradation of particular homologues belonging to Lutensol GD 70 at 120 mg L^−1^ subjected to the test. Only the biodegradation of C_4_Glu was higher, while for the rest of the homologues, the biodegradation was lower or the same, like for the sample without diesel oil. A considerable difference was observed for the sample containing 360 mg L^−1^ of Lutensol GD 70 with diesel oil degraded by the stressed Ps9 bacterial strain. Although the results for C_10_Glu_1-4_ homologues were similar to those obtained at 120 mg L^−1^ of Lutensol GD 70, considerably lower biodegradation was noted for C_4_Glu_1-4_ homologues with shorter alkyl chain. Nevertheless, it can be concluded that the stress put on bacterial strain has greater influence on the biodegradation of Lutensol GD 70 than both concentration of Lutensol GD 70 and the addition of diesel oil.

No publications about Lutensol GD 70 biodegradation were found. Jurado et al. (2011) investigated biodegradation of another alkyl polyglucoside, Glucopon 650. In 7-day cultures inoculated with an active sludge, the biodegradation of surfactant was about 60 % at concentrations less than 50 mg · L^-1^. But at the concentration 75 mg · L^-1^ and 100 mg · L^-1^ the biodegradation was about 10 %. Zgoła-Grześkowiak et al. (2008) using modified OECD screening test, observed after one week nearly 100 % biodegradation of all fractions of Glucopon 215 and Glucoon 600. Qin et al. (2006) using activated sludge obtained about 90 % biodegradation of short chained (C8 and C10) fractions. Our results have shown that better results of surfactant biodegradation were obtained for the strain with long-term contact with diesel oil.

## Conclusions

Alkyl polyglucosides, such as Lutensol GD 70, can be applied in the environment as a factor supporting the bioavailability of hydrophobic carbon sources. It is really biodegradable by microorganisms. This surfactant in different way modifies cell surface of tested strains. Adding it to the diesel oil system resulted in an increase of cell surface hydrophobicity and the formation of cell aggregates (a high polydispersity index). Moreover, the correlation between surfactant concentrations and cell surface hydrophobicity was not observed. Furthermore, a long-term contact with diesel oil (24 months) modifies not only cell surface properties of tested strain but also its ability of surfactant biodegradation.
